# Metformin alters therapeutic effects in the BALB/c tumor therapy model

**DOI:** 10.1186/s12885-021-08354-x

**Published:** 2021-05-28

**Authors:** Felix B. Meyer, Sophie Goebel, Sonja B. Spangel, Christiane Leovsky, Doerte Hoelzer, René Thierbach

**Affiliations:** Friedrich-Schiller-Universität Jena, Fakultät für Biowissenschaften, Institut für Ernährungswissenschaften, Abteilung Humanernährung, Jena, Germany

**Keywords:** Metformin, Cancer, In vitro model, Adjuvant, Energy metabolism

## Abstract

**Background:**

Despite considerable medical proceedings, cancer is still a leading cause of death. Major problems for tumor therapy are chemoresistance as well as toxic side effects. In recent years, the additional treatment with the antidiabetic drug metformin during chemotherapy showed promising results in some cases. The aim of this study was to develop an in vitro tumor therapy model in order to further investigate the potential of a combined chemotherapy with metformin.

**Methods:**

Cytotoxic effects of a combined treatment on BALB/c fibroblasts were proven by the resazurin assay. Based on the BALB/c cell transformation assay, the BALB/c tumor therapy model was established successfully with four different and widely used chemotherapeutics from different categories. Namely, Doxorubicin as a type-II isomerase inhibitor, Docetaxel as a spindle toxin, Mitomycin C as an alkylating agent and 5-Fluorouracil as an antimetabolite. Moreover, glucose consumption in the medium supernatant was measured and protein expressions were determined by Western Blotting.

**Results:**

Initial tests for the combined treatment with metformin indicated unexpected results as metformin could partly mitigate the cytotoxic effects of the chemotherapeutic agents. These results were further confirmed as metformin induced resistance to some of the drugs when applied simultaneously in the tumor therapy model. Mechanistically, an increased glucose consumption was observed in non-transformed cells as well as in the mixed population of malignant transformed cell foci and non-transformed monolayer cells, suggesting that metformin could also increase glucose consumption in transformed cells.

**Conclusion:**

In conclusion, this study suggests a cautious use of metformin during chemotherapy. Moreover, the BALB/c tumor therapy model offers a potent tool for further mechanistic studies of drug-drug interactions during cancer therapy.

**Graphical abstract:**

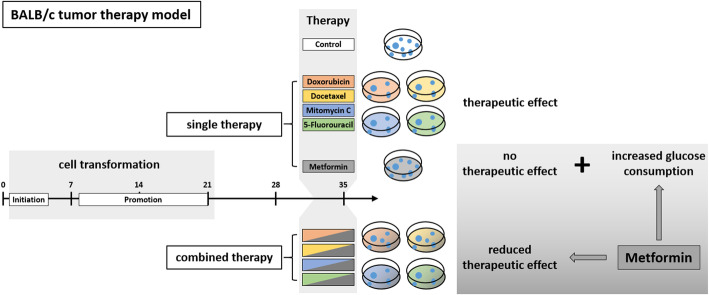

**Supplementary Information:**

The online version contains supplementary material available at 10.1186/s12885-021-08354-x.

## Background

Worldwide, cancer is one of the leading causes of death with estimated 9.6 million deaths in 2018 [1]. Due to medical proceedings, the survival rates are increasing but chemoresistance and toxic side effects are still major problems for chemotherapy. Thereby, the combined treatment of chemotherapeutics with several substances seems to be a promising approach [2]. In 2005, it was demonstrated for the first time that diabetic patients taking the widely used antidiabetic drug metformin show lower incidences for developing cancer [3]. Since then, the anticancer effects of metformin got into the focus of cancer research.

Molecularly, metformin inhibits the mitochondrial complex I following an increase in the ATP:AMP ratio that finally leads to an activation of the cellular energy sensor 5′ AMP-activated protein kinase (AMPK) [4]. As a result, metformin reduces blood glucose levels systemically via AMPK-mediated inhibition of hepatic gluconeogenesis [5–7] and an increased glucose uptake in peripheral tissues [8, 9], both leading to lower insulin levels consequently. This could partly explain the anticancer effects of metformin, since increased glucose and insulin levels are associated with cancer proliferation and mortality [10, 11].

In addition, metformin exerts direct effects on cancer cells and is able to reduce glucose consumption via reversion of the Warburg effect in several tumor cell lines independent of AMPK [12–16]. Therefore, metformin is discussed as an adjuvant in tumor therapy in diabetic and non-diabetic patients with promising results especially for colorectal and prostate cancer [17, 18]. For the use of several chemotherapeutic agents, toxic side effects are a dose-limiting factor that could also be improved by metformin. For example, the cardiotoxicity of Doxorubicin (Dox) is reduced when the treatment is combined with metformin [19]. Moreover, metformin could enhance the effectiveness of Docetaxel (Dtx) in hyperglycemic conditions [20], suggesting its promising role for cancer treatment in patients with diabetes.

Even though several anticarcinogenic effects of metformin are observed, the clinical data are still contentious depending on disease-related (type of tumor, clinical stage, form of treatment) and on patient-related factors (insulin resistance, age, sex) [21]. For in vitro experiments, the combination of metformin with several chemotherapeutic agents shows controversial results ranging from synergistic effects [22–24] to even adverse effects [25, 26]. So far, these investigations underline the need for a more detailed understanding of the molecular mechanisms that occur when combining metformin with chemotherapeutics before applying it as a potential adjuvant in chemotherapy.

A helpful tool could be the BALB/c cell transformation assay (BALB-CTA) which mimics different phases of malignant cell transformation in vitro and is eligible for mechanistic cancer research [27]. With an additional treatment during the assay, the potential effects of different chemotherapeutic agents can be investigated and molecular mechanisms further analyzed [28]. In the present study, we intended to establish a BALB/c tumor therapy model (BALB-TTM) using commonly applied chemotherapeutic agents. Thereby, the treatment was conducted during the late phase of malignant cell transformation on already existing cell foci. In a second step, the combined therapy of metformin with the chemotherapeutic agents was tested. Thus, the BALB-TTM could be a strong tool for clarifying molecular mechanisms of drug-drug interactions and the development of more effective chemotherapies.

## Methods

### Cell culture

BALB/c-3 T3-A31-1-1 cells from Hatano Research Institute of Japan were kindly provided by Dr. A. Poth (Harlan Cytotest Cell Research GmbH, Roßdorf) and used for all of the experiments. Cells were cultivated with DMEM/HAM’s F-12 (Biochrom #T481) containing 3.15 g/l D-glucose, 5% fetal bovine serum and 1% penicillin/streptomycin in an incubator (37 °C, 5% CO_2_, 95% humidity). Only subconfluent grown cells (70 to 80% confluence) between the passages 20 to 45 were used. Tests for mycoplasmas were conducted regularly and were negative.

### Cell viability assay

The indicator dye alamarBlue® (Bio-Rad #BUF012) was used to determine cell viability. Cells were seeded in 96 well plates (15,000 per well) and allowed to grow confluent for 48 h. Afterwards, cells were treated for 24 h with the chemotherapeutics, metformin or a combination of both. In every treatment group, the DMSO concentration was adapted to a constant level of 0.1%. Finally, medium was discarded and non-fluorescent alamarBlue® containing medium (ratio 1:10) was added. Fluorescence signal was measured after 0 h (blank) and 3 h (Ex 540 nm/Em 590 nm). Thereby, the reduction of non-fluorescent resazurin to fluorescent resorufin is proportional to cell viability. The cell viability assay was performed with 6 technical replicates for the single treatment and with 3 technical replicates for the combination therapy in 3 biological replicates, respectively.

### BALB/c cell transformation assay

The BALB-CTA was performed according to the recommended protocol of the European Centre for the Validation of Alternative Methods [29]. Differing from this protocol, cells were cultivated for the whole time in DMEM/HAM’s F-12 medium and the duration of the assay was prolonged to 42 days. On the first day, 5000 cells per well were seeded in Corning® Primaria™ 6-well-plates (Corning #353846) and cultivated under standard conditions. Change of medium was performed twice a week. Malignant cell transformation was induced by treatment with the tumor initiator 3-methylcholanthrene (MCA, Sigma #213942) (0.5 μg/ml, dissolved in DMSO) from day 1–4 following the tumor promotor 12-tetradecanoylphorbol-13-acetate (TPA, Sigma #79346) (0.3 μg/ml, dissolved in DMSO) from day 8–21. Consequently, cells lose their contact inhibition and start to grow over the monolayer and as a result, characteristic cell foci of transformed cells are formed. An additional treatment was conducted either chronically from day 1–42 or therapeutically from day 32–42 with metformin (for details see Fig. 2a). On day 42, cells were washed twice with cold (4 °C) PBS and fixed with cold PBS/methanol (ratio 1:1) for 3 min following treatment with ice cold methanol (− 20 °C) for 10 min. Finally, cells were washed twice with ice cold methanol and dried at room temperature. For better visualization of cell foci, cells were stained with Giemsa solution (AppliChem #251338). Per well, 1 ml Giemsa solution was added, incubated for 3 min and afterwards diluted with 3 ml of deionized water and incubated for further 3 min. The whole solution was discarded and cells were washed 5 times with tap water following 5 × 10 min washing with deionized water on the plate shaker.

In order to establish the BALB-TTM, cells were treated from day 32–35 with the chemotherapeutics Doxorubicin (Cayman #15007, dissolved in DMSO), Docetaxel (Cayman #11637, dissolved in DMSO), Mitomycin C (Fisher Scientific #10182953, dissolved in DMSO), 5-Fluorouracil (Sigma-Aldrich #F6627, dissolved in DMSO), the antidiabetic drug metformin (Sigma-Aldrich #PHR1084, dissolved in water) or with the chemotherapeutics in combination with metformin. Due to the high potency of the chemotherapeutic drugs, cells were fixed already on day 35. For all experiments, DMSO served as a solvent control and was adapted to a constant concentration of 0.05% from day 1–32. The treatment with the chemotherapeutics on day 32–35 increased the DMSO concentration that was now kept constant at 0.1%. Unless stated otherwise, the assays were performed with 4 technical replicates in 4 biological experiments. The number of type-III foci was counted independently by 2 different people as described elsewhere [30].

### Glucose measurement

Glucose concentration was determined in medium supernatant using medium without phenol red. Cells were seeded in 10 cm cell culture dishes (600,000 cells/dish) and allowed to grow confluent for 72 h. Cell monolayer was treated with 1 mM and 10 mM metformin and 1 ml of cell culture supernatant was collected after 0, 24, 48, 72 and 96 h. Samples were diluted 1:15 or 1:45 with deionized water. Standard series with 0, 10, 20, 40, 60 and 80 μg/ml was generated with deionized water and D-Glucose solution (Sigma-Aldrich #G3285). One capsule of glucose oxidase/peroxidase reagent (Sigma-Aldrich #G3660) was solved in 39.2 ml deionized water and a stock solution with 5 mg/ml of o-dianisidine dihydrochloride (Sigma-Aldrich #F5803) was prepared. Finally, the two components were mixed in the ratio 1:50 in order to generate the assay reagent. Probes and standards were pipetted in quadruples on a 96 well plate (60 μl/well), the assay reagent was added (120 μl/well) and incubated for 30 min at 37 °C. The oxidation of glucose to gluconic acid via the glucose oxidase generates hydrogen peroxides that further react with o-dianisidine in presence of the peroxidase to form a brown colored product. By adding 120 μl/well 6 M sulfuric acid (Carl Roth #4623.1) the reaction stops and a stable pink colored product is formed. The intensity of the pink color can be measured at 540 nm and is proportional to the initial glucose concentration. Glucose measurement was conducted in 3 biological replicates.

### Protein extraction and immunoblot

Cells were harvested with cell lysis buffer (Cell Signaling #9803) and sonicated (UP200S, Hielscher Ultrasonics GmbH) afterwards. Proteins were isolated after centrifugation and concentration was determined according to the Bradford method [31]. SDS-PAGE was performed with a 10% gel using 30 μg protein per lane. Proteins were transferred on a nitrocellulose membrane with the semi-dry Western Blot and incubated with phospho-AMPK (Cell Signaling #2535), AMPK (Cell Signaling #2532) or α-Tubulin (Sigma Aldrich #T9026) following the secondary antibodies anti-Rabbit (Cell Signaling #7074) or anti-Mouse (Cell Signaling #7076).

### Statistical analysis

The software IBM SPSS was used for all statistical analyses. The results were tested for homogeneity of variances each and as described elsewhere [32, 33], the normal distribution was neglected. For the cell viability assay, a one-way ANOVA was performed following the Dunnett-T post-hoc test in case of homogeneity of variances or the Dunnett-T3 if this was not the case. Statistical differences of the type-III foci in the BALB-TTM and the glucose concentration in the medium were calculated for existing homogeneity of variances with a one-way ANOVA and an additional Bonferroni post-hoc test. Otherwise, a Dunnett-T3 post-hoc test was performed.

Positive or negative drug combination effects were further described with the Highest Single Agent approach [34]. According to that, the Combination Index (CI) was calculated as following, with max(E_A_,E_B_) describing the effect of the highest concentration of the single agent and E_AB_ for the effect for the combination treatment:

$$ CI=\frac{\max \left({E}_A,{E}_B\right)}{E_{AB}} $$

Hence, the CI gives information whether the combination of two components shows greater (CI > 1) or smaller effects (CI < 1) compared to a single agent alone.

## Results

### Metformin affects glucose consumption in non-transformed BALB/c cells

**Fig. 1:**
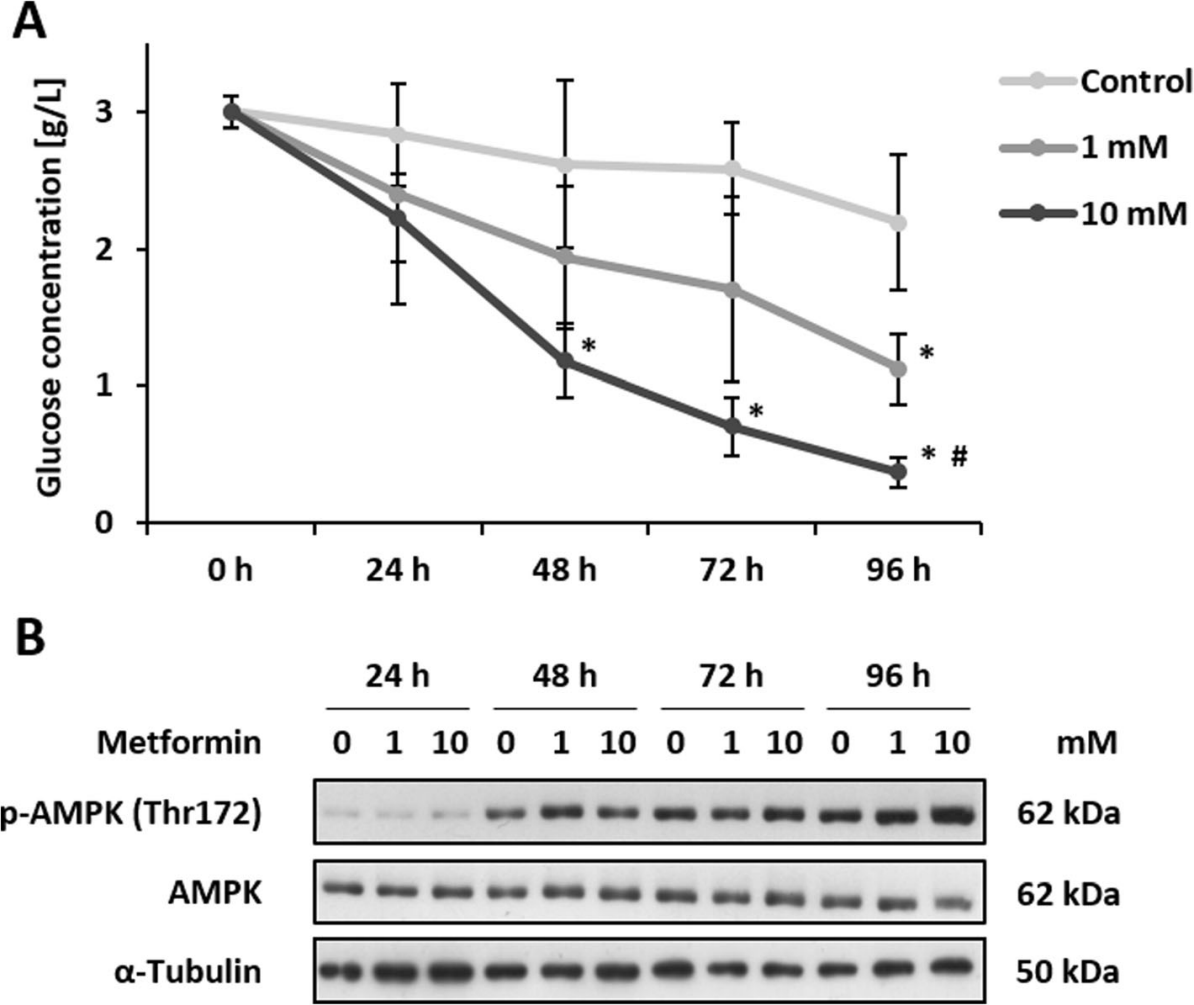
Altered energy metabolism after metformin treatment. Non-transformed BALB/c cells were seeded in 10 cm cell culture dishes and allowed to grow confluent for 72 h. Afterwards, cells were treated with 1 or 10 mM metformin. Medium supernatant was collected after 0, 24, 48, 72 and 96 h and cells were harvested for protein analysis. **a** Glucose concentration was measured and data are shown as mean + SD of 3 biological replicates. Statistical differences were calculated with a one-way ANOVA (post-hoc: Bonferroni or Dunnett-T3) with * = (*p* < 0.05) vs. control and # = (*p* < 0.05) vs. 1 mM metformin for each point in time. **b** Proteins were extracted and protein expression as well as phosphorylation levels of AMPK at Thr172 were detected via immunoblot in 3 biological replicates. After detection of p-AMPK, the membrane was stripped two times and re-probed with AMPK mAB and α-Tubulin mAB to confirm equal loading. Images shown are cropped from full-length blots represented in Additional file 2: Supplementary Figure 2

A potential influence of metformin on energy metabolism was investigated first. In control cells, the glucose concentration decreased steadily from 3.15 g/l reaching 2.2 g/l after 96 h. Metformin increases glucose consumption significantly and dose-dependently. After 24 h treatment, glucose concentration was already at 2.4 and 2.2 g/l for 1 and 10 mM metformin, respectively. Incubation with metformin for 96 h leads to glucose concentrations of 1.1 and 0.4 g/l for 1 and 10 mM (Fig. 1a). Phosphorylation levels of AMPK increased over time but interestingly, metformin showed no consistent effect on both, phosphorylation and expression of AMPK (Fig. 1b). However, the densitometric analysis indicates an increased phosphorylation level of AMPK after the treatment with 10 mM metformin for 96 h associated with a lower protein level of AMPK (Additional file 1: Supplementary Figure 1). This could be due to a lack of nutrients, especially glucose.

### Anticarcinogenic effects of metformin in the BALB-CTA

**Fig. 2:**
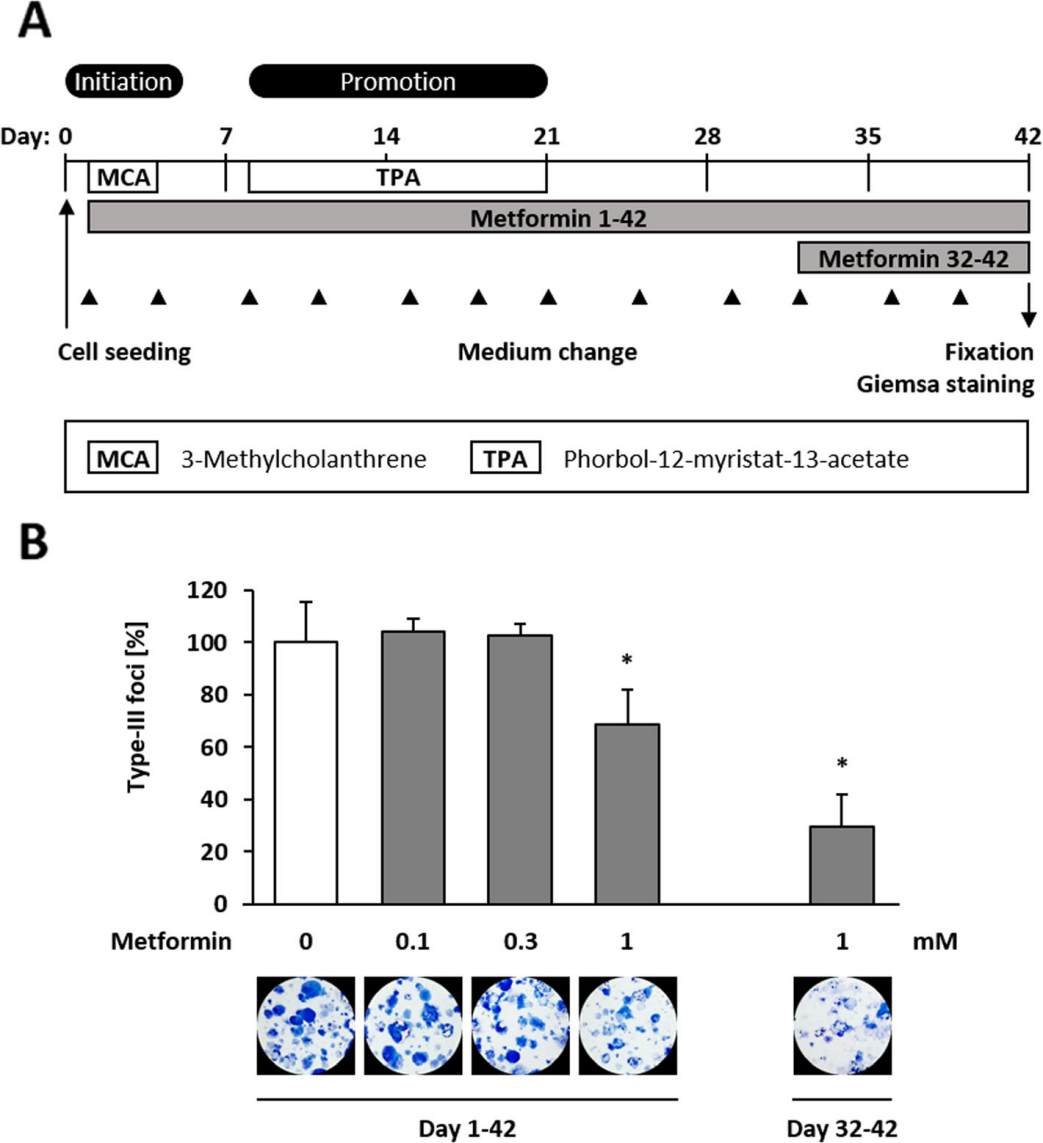
Anti-carcinogenic effect of Metformin in the BALB-CTA. **a** The BALB/c 3 T3 cell transformation assay was performed according to the recommended protocol. In brief, cells were seeded in 6-well plates and treated with the tumor initiator MCA (0.5 μg/ml) on day 1–4 and the tumor promotor TPA (0.3 μg/ml) from day 8–21 in order to induce malignant cell transformation. Metformin was added additionally either chronical from day 1–42 or in the late phase of malignant cell transformation from day 32–42. On day 42, cells were fixed with methanol and stained with Giemsa solution for better visualization of cell foci. **b** Representative pictures and the number of type-III foci of 3 biological replicates (mean + SD) are shown. Statistical differences were calculated with a one-way ANOVA (post-hoc: Bonferroni) with * = (*p* < 0.05) vs. control

In order to study the effects of metformin on malignant cell transformation, a BALB-CTA was performed as described earlier [27, 28] (Fig. 2a). Permanent treatment (day 1–42) with 1 mM metformin showed anticarcinogenic effects and decreased the number of type-III foci significantly by 32% while lower concentrations had no effect. Furthermore, we also observed an effect when 1 mM metformin was added for a shorter duration from day 32 until day 42 on the cell monolayer with already existing cell foci as the number of type-III foci decreased by 70% (Fig. 2b).

### Establishment of the BALB/c tumor therapy model (BALB-TTM)

**Fig. 3:**
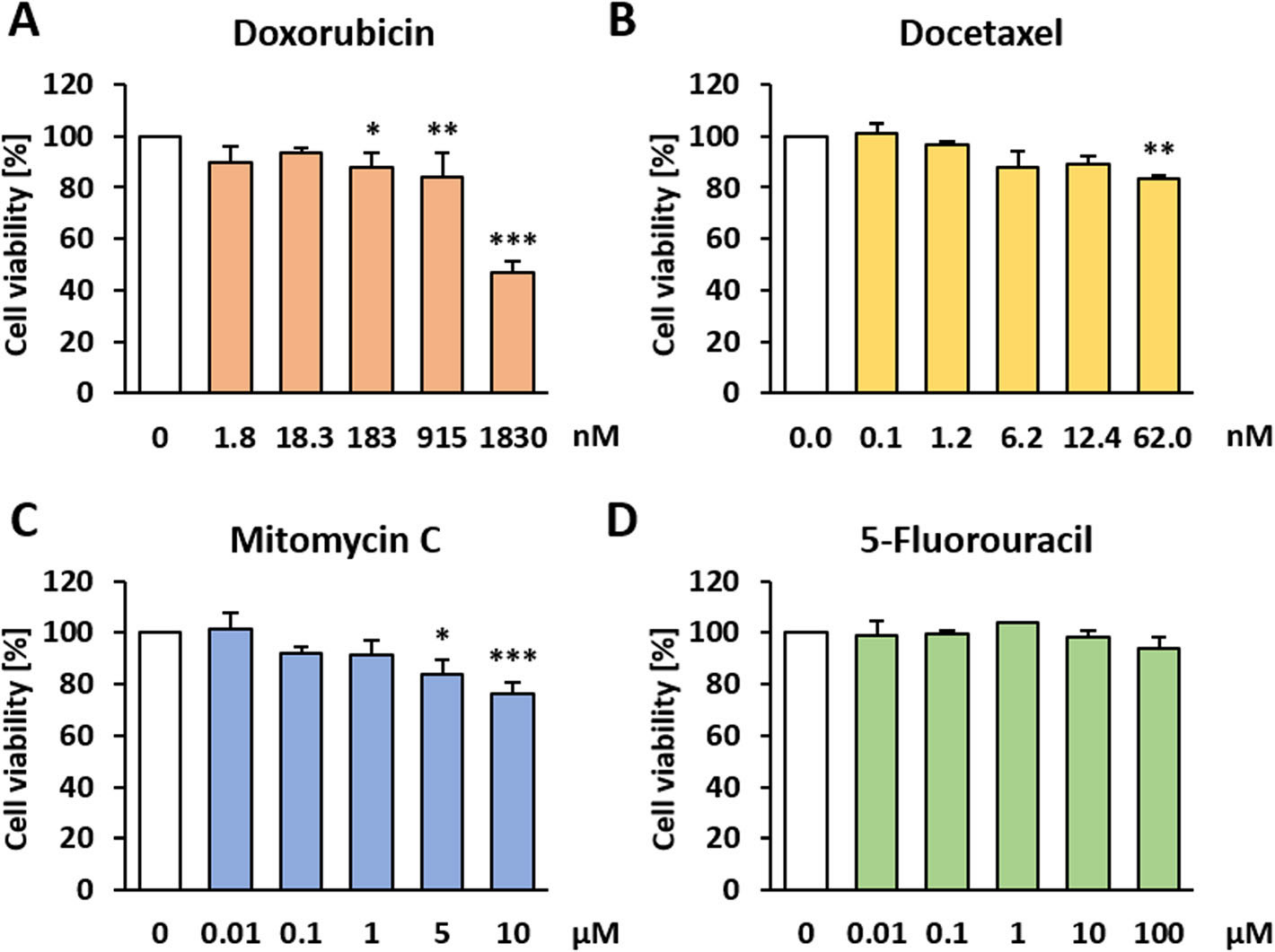
Cytotoxic effects of chemotherapeutics on BALB/c cells. Non-transformed BALB/c cells were seeded in 96 well plates and allowed to grow confluent for 48 h. Afterwards, cells were treated for 24 h with different concentrations of **a** Doxorubicin, **b** Docetaxel, **c** Mitomycin C or **d** 5-Fluorouracil. Cell viability was measured indirectly by the reduction of resazurin to fluorescent resorufin. Data are shown as mean + SD of 3 biological replicates. Statistical differences were calculated with a one-way ANOVA (post-hoc: two sided Dunnett-T or Dunnett-T3) with * = (*p* < 0.05); ** = (*p* < 0.01) and *** = (*p* < 0.001) vs. control

**Fig. 4:**
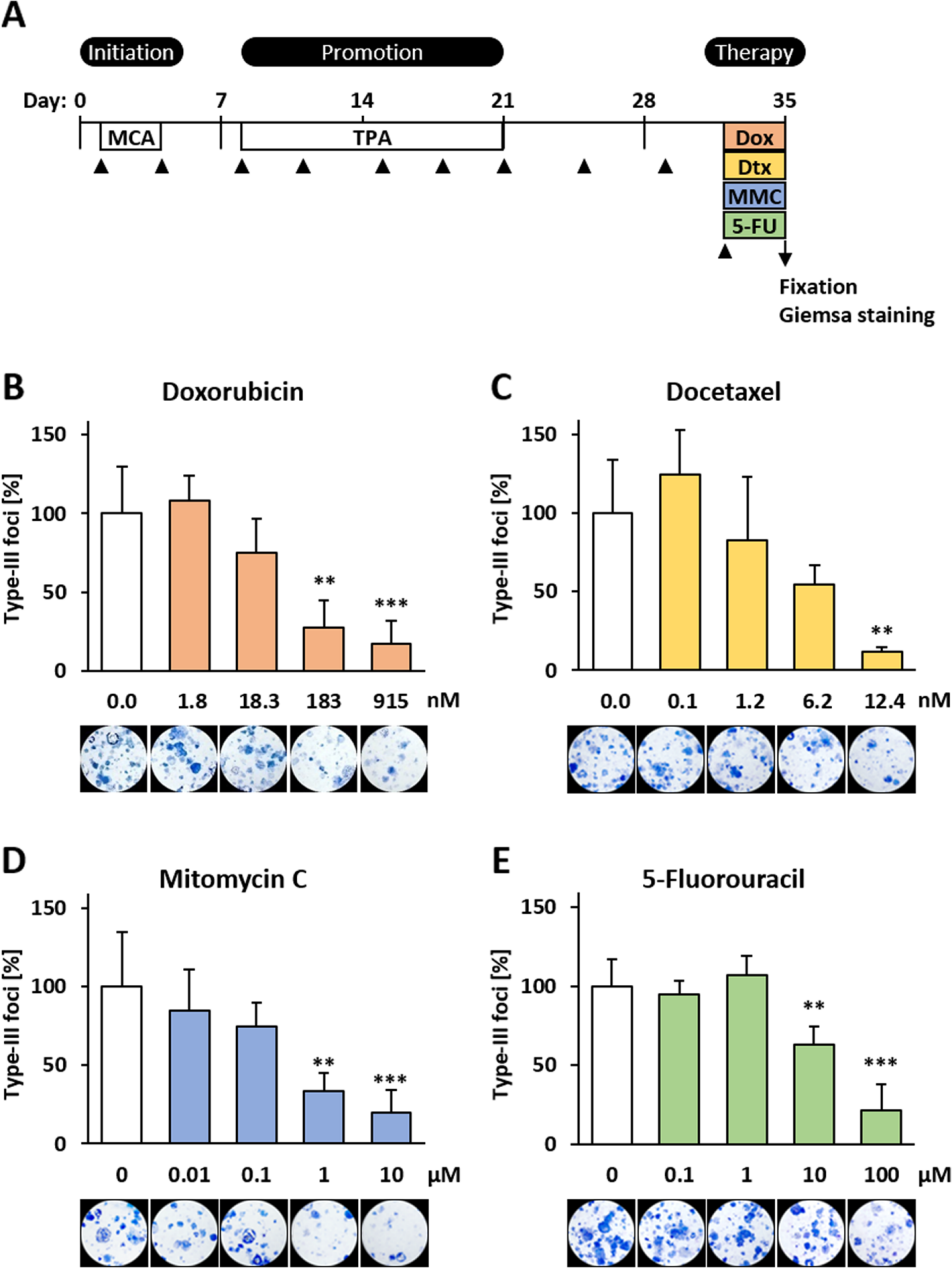
Establishment of the BALB/c tumor therapy model (BALB-TTM) with chemotherapeutic agents. **a** The BALB/c 3 T3 cell transformation assay was performed as described earlier. An additional treatment was conducted on day 32 with Doxorubicin (Dox), Doxetaxel (Dtx), Mitomycin C (MMC) or 5-Fluorouracil (5-FU) for 72 h and cells were fixed on day 35. Representative pictures and the number of type-III foci of 3 biological replicates (mean + SD) are shown for different concentrations of **b** Doxorubicin, **c** Docetaxel, **d** Mitomycin C and **e** 5-Fluorouracil. Statistical differences were calculated with a one-way ANOVA (post-hoc: Bonferroni or Dunnett-T3) with ** = (*p* < 0.01) and *** = (*p* < 0.001) vs. control

**Fig. 5:**
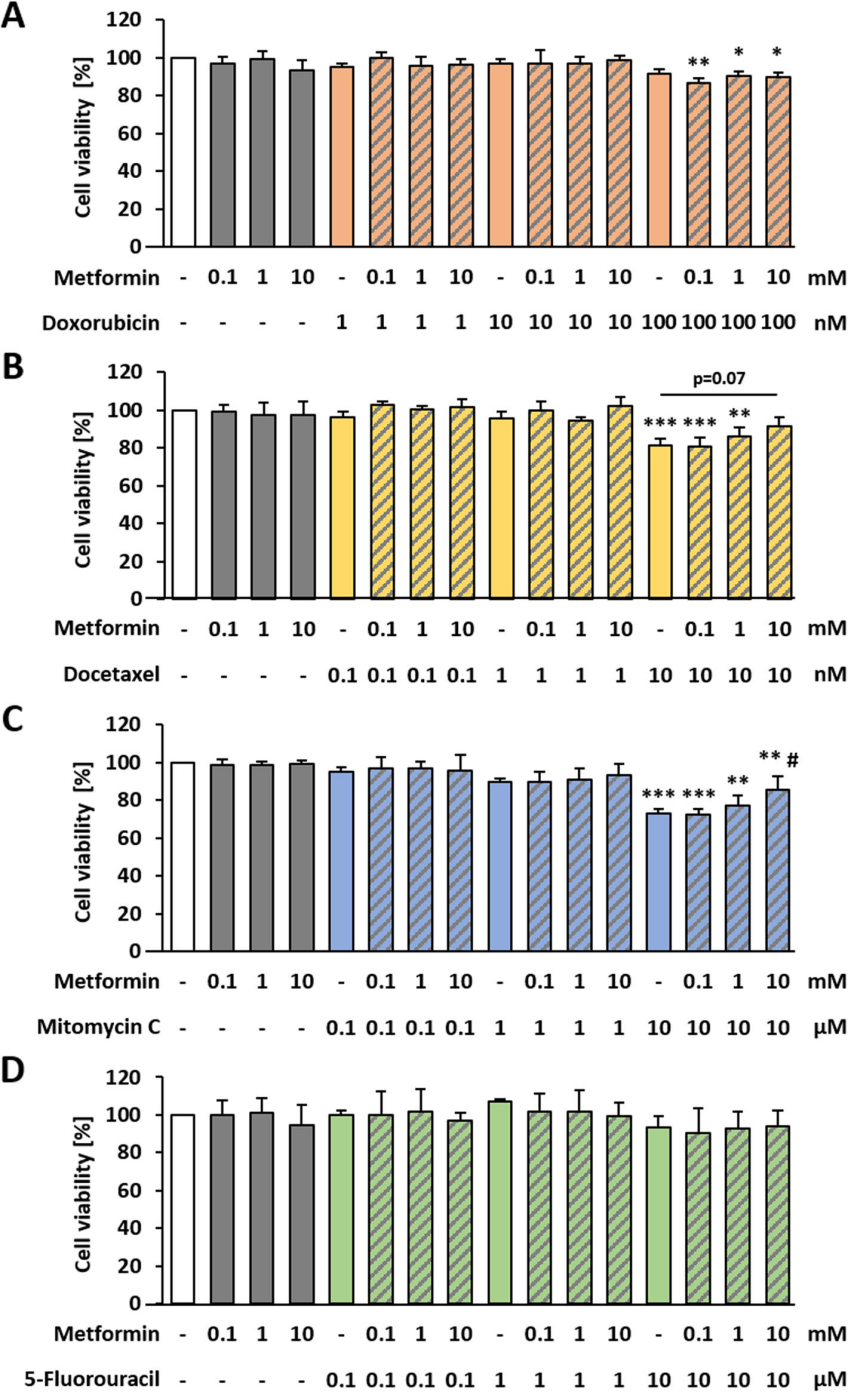
Cytotoxic effects of chemotherapeutics plus metformin on BALB/c cells. Non-transformed BALB/c cells were seeded in 96 well plates and allowed to grow confluent for 48 h. Afterwards, cells were treated for 24 h with different concentrations of **a** Doxorubicin, **b** Docetaxel, **c** Mitomycin C or **d** 5-Fluorouracil alone or in combination with different concentrations of metformin. Cell viability was measured indirectly by the reduction of resazurin to fluorescent resorufin. Data are shown as mean + SD of 3 biological replicates. Statistical differences were calculated with a one-way ANOVA (post-hoc: two sided Dunnett-T or Dunnett-T3) with * = (*p* < 0.05); ** = (*p* < 0.01) and *** = (*p* < 0.001) vs. control and # = (*p* < 0.05) vs. 10 μM Mitomycin C

Because treatment in the late phase of the BALB-CTA is comparable to a therapeutic usage, we asked whether the BALB-CTA is suitable for therapy questions in general. Therefore, the applicability of a BALB-TTM was tested by using chemotherapeutic agents with different mode of actions. Namely, Dox as a type-II isomerase inhibitor, Mitomycin C (MMC) as an alkylating agent, 5-Fluorouracil (5-FU) as an antimetabolite and Dtx as a spindle toxin. Suitable concentrations were determined prior with the Resazurin cell viability assay on non-transformed BALB/c cells. Cell viability was decreased significantly after treatment with 183 nM Dox by 12% (Fig. 3a), 62 nM Dtx by 17% (Fig. 3b) and 5 μM MMC by 16% (Fig. 3c). 5-FU showed no cytotoxic effects at a concentration up to 100 μM (Fig. 3d). For the BALB-TTM establishment, a BALB-CTA was performed with following modifications. Because of the high potency of the chemotherapeutic drugs, the therapeutic treatment was shortened to 72 h from day 32 to 35 (Fig. 4a). After the treatment in the late phase of malignant cell transformation, a significantly reduced number of type-III foci was observed for all of the tested chemotherapeutic agents. With the exception of Dox (Fig. 4b), this was even the case for non-toxic concentrations, namely 12.4 nM Dtx (Fig. 4c), 1 μM MMC (Fig. 4d) and 10 μM 5-FU (Fig. 4e).

### Effects of chemotherapeutic agents plus metformin on non-transformed cells

In order to get suitable concentrations for the combined therapy with metformin plus chemotherapeutic agents in the BALB-TTM, the cell viability of non-transformed BALB/c cells was measured. The single treatment with 0.1, 1 and 10 mM metformin had no impact on cell viability (Fig. 5a-d). Up to 100 nM Dox showed no effects on cell viability but it was decreased significantly after the combined treatment with 100 nM Dox plus 0.1 mM metformin by 14% (CI = 1.06), plus 1 mM metformin by 10% (CI = 1.01) and plus 10 mM metformin by 10%(CI = 1.02), respectively (Fig. 5a). Dtx led to a significant decrease in cell viability at a concentration of 10 nM by 19%. However, the supplementary treatment with 10 mM metformin abolished this effect. Thus, the observed decrease in cell viability was only at 9% (CI = 0.89) (Fig. 5b), meaning that metformin seems to protect the cells. A cytotoxic effect for MMC was detected at a concentration of 10 μM, decreasing cell viability by 27%. The additional treatment with 10 mM metformin increased cell viability significantly (CI = 0.85) but was still cytotoxic with a decrease in cell viability by 15% (Fig. 5c). Neither the single treatment with 5-FU nor the combination with metformin showed any effect on cell viability (Fig. 5d).

### Combined treatment in the BALB-TTM

**Fig. 6:**
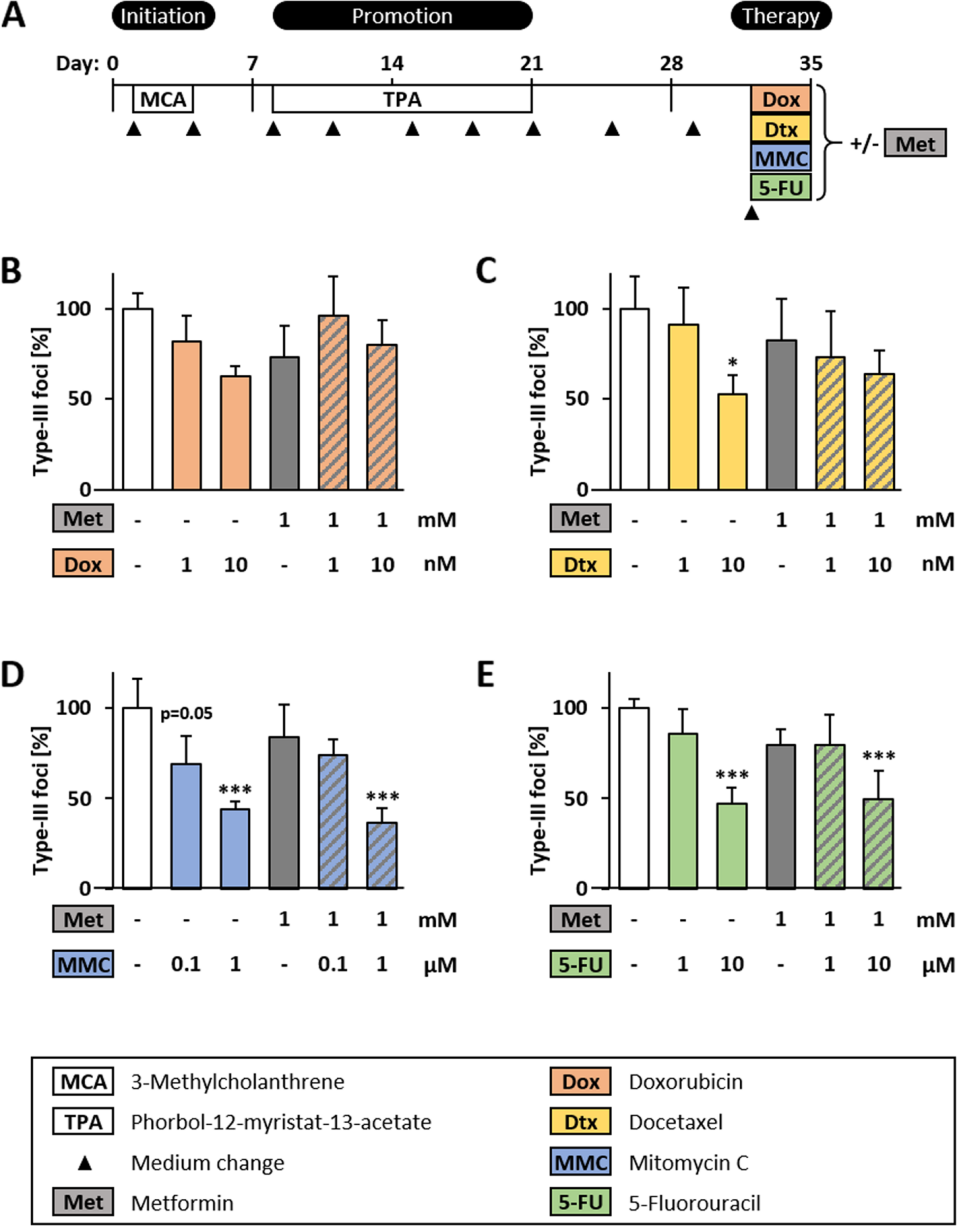
Combined treatment with chemotherapeutic agents plus metformin in the BALB-TTM. **a** The BALB/c 3 T3 cell transformation assay was performed as described earlier. An additional treatment was conducted on day 32 with Doxorubicin, Doxetaxel, Mitomycin C or 5-Fluorouracil alone or in combination with metformin for 72 h and cell were fixed on day 35. The number of type-III foci of 4 biological replicates (mean + SD) are shown for different concentrations of **b** Doxorubicin, **c** Docetaxel, **d** Mitomycin C and **e** 5-Fluorouracil in combination with 1 mM metformin. Statistical differences were calculated with a one-way ANOVA (post-hoc: Bonferroni) with * = (*p* < 0.05) and *** = (*p* < 0.001) vs. control

The effectiveness of a combined therapy with chemotherapeutic agents plus metformin was tested in the BALB-TTM (Fig. 6a). For all chemotherapeutics, two concentrations were chosen and tested alone or in combination with 1 mM metformin. According to the preliminary tests, the lower concentration should not reduce number of type-III foci and the higher concentration reduces it significantly. Treatment with 1 mM metformin alone in the BALB-TTM decreased number of type-III foci up to 27% but this effect was not significant (Fig. 6b-e). For Dox, a therapeutic effect was observed only with the toxic concentrations of 183 nM and 915 nM (Fig. 4b). Therefore, 1 and 10 nM Dox, which show no significant effect on type-III foci, were combined with metformin. Compared to the single treatments with the chemotherapeutics, the co-treatment increased the number of type-III foci by 14% (CI = 0.86) and 18% (CI = 0.78) but this effect was not significant (Fig. 6b). The lower concentration of 1 nM Dtx showed no therapeutic effect in the BALB-TTM and 10 nM reduced number of type-III foci significantly by 48%. However, the combination with metformin neglected this effect, meaning that 10 nM Dtx plus 1 mM metformin showed a 12% smaller and no significant decrease in the number of type-III-foci (CI = 0.82) (Fig. 6c). For MMC and 5-FU, the lower concentrations of 0.1 μM or 1 μM had no significant effect on the number of type-III foci and the higher concentrations of 1 μM or 10 μM reduced it significantly by 56% for MMC and by 53% for 5-FU. The co-treatment with metformin showed the same results as for the single treatment. Only the higher concentrations combined with 1 mM metformin could reduce the number of type-III foci comparable to the single treatment (CI = 1.19 for MMC and 0.95 for 5-FU) (Fig. 6d+e).

### Metformin affects glucose consumption in the BALB-TTM

**Fig. 7:**
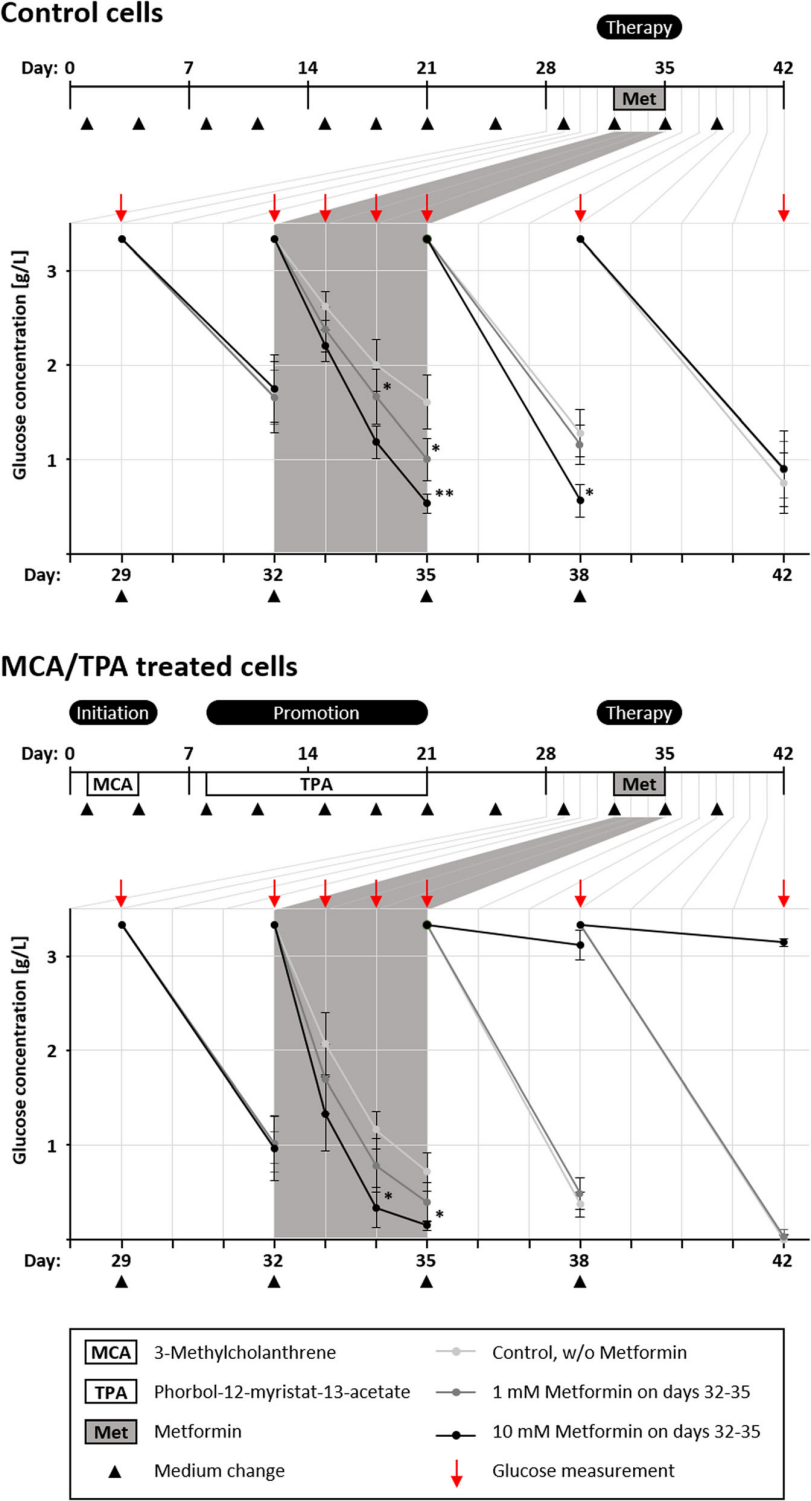
Metformin alters glucose consumption in the BALB-TTM. The BALB/c 3 T3 cell transformation assay was performed as described earlier. An additional treatment was conducted from day 32–35 with metformin. On day 35 and 38, fresh medium without metformin was added again. Control cells were not treated with MCA/TPA. Medium supernatant was collected at day 32, 33, 34, 35, 38 and 42 and glucose concentration was measured. The slanting lines indicate the decrease of glucose after fresh medium with 3.15 g/l D-glucose was added every 3–4 days. Statistical differences were calculated with a one-way ANOVA (post-hoc: Bonferroni) with * = (*p* < 0.05) and ** = (*p* < 0.01) vs. control for each point in time

Because metformin alters glucose metabolism in non-transformed BALB/c cells, we measured the glucose concentration in the medium supernatant at different points in time of the BALB-TTM as a first mechanistical analysis. As a control, we cultivated cells without treatment of MCA/TPA so that only a monolayer of non-transformed cells was formed. According to the protocol of the BALB-TTM, the cells were treated with metformin on day 32 for 72 h and the glucose concentration was measured after 24, 48 and 72 h. In order to investigate adaptive effects, we further cultivated the cells until day 42 and measured the glucose concentration after 3 or 4 days before fresh medium was added (Fig. 7). Glucose consumption was in the same range in every well before metformin was added with glucose concentrations between 1.66 and 1.75 g/l in the DMSO treated cells and 0.97 and 1.01 g/l in the MCA-TPA treated cells after 3 days (day 29 to 32). As expected, 1 mM and 10 mM metformin increased glucose consumption significantly in the monolayer cells (day 32–35). Compared to the control cells with 1.60 g/l on day 35, metformin led to glucose concentrations of 1.00 g/l and 0.53 g/l for 1 mM and 10 mM, respectively. In the MCA-TPA treated cells, where a mixed culture of malignant transformed, foci forming cells and the non-transformed monolayer cells exists, metformin again increased glucose consumption significantly at a concentration of 10 mM reaching saturation on day 35. Compared to the control cells with 1.16 g/l on day 34, metformin led to glucose concentrations of 0.78 g/l and 0.34 g/l for 1 mM and 10 mM, respectively. An adaptive effect was observed for 10 mM metformin in the non-transformed monolayer cells where glucose consumption was increased also on day 38 when cells were no longer treated with metformin. In the mixed culture of non-transformed and malignant transformed cells, 10 mM metformin decreased the glucose concentration to a minimum after 72 h. Probably, the lacking glucose in the medium induced cell death and finally stops glucose consumption, leading to a stable glucose concentration in the medium from day 35–42 (Fig. 7).

## Discussion

In the present study, we modified the well-known BALB-CTA by adding an additional treatment on day 32 for 72 h in order to establish an in vitro tumor therapy model, the BALB-TTM. The effectiveness was proven successfully with 4 well-established chemotherapeutic agents and furthermore, for the very first time, a combined treatment with metformin was tested. The results are surprising as they show that metformin could partly mitigate the effects of the chemotherapeutic agents and a deregulated glucose metabolism seems to be involved in this process.

In vitro cell transformation assays mimic different phases of the in vivo multi-step carcinogenesis process. They are used by chemical, cosmetic and pharmaceutical industries for more than 6 decades to screen agents for carcinogenicity [35]. We have shown previously that the BALB-CTA is also combinable with different molecular biologic and biochemical methods, thus allowing to screen for molecular mechanisms [27, 28]. In this case, the malignant cell transformation is induced by treatment with the tumor initiator MCA following the tumor promotor TPA. Consequently, transformed cells lose their contact inhibition, start to grow over the monolayer of non-transformed BALB/c cells and pile up to characteristic, multilayered cell foci. For the BALB-TTM, an additional therapeutic treatment was performed on day 32 for 72 h on already existing cell foci. A reduction of the number of type-III foci could hence indicate a chemotherapeutic potential of the tested substance. Compared to rodent studies, this assay is less time consuming, needs a lower amount of resources and has no ethical implications. Moreover, molecular modes of action could be investigated easily, standardized and compared between non-transformed and malignant transformed cells.

The anticancer effects of metformin are widely described in vitro and in vivo [36] and now, were also confirmed in the BALB-CTA. Comparable to diabetic patients who show lower incidences for developing cancer when taking metformin for years [3, 37], chronical treatment with 1 mM metformin decreases number of type-III foci significantly and shows a tumor preventive effect. At this point, the BALB-CTA offers a strong tool for further mechanistic studies. Moreover, when metformin was added in the late phase of the BALB-CTA on already existing cell foci, a chemotherapeutic effect was observed with a significant decrease in number of type-III foci. Although plasma concentrations of metformin in diabetic patients are in the lower range of 10 to 40 μM [38], it was shown that metformin accumulates highly in tissues of mice, especially in the gastrointestinal tract where concentrations were up to 50 times higher compared to plasma but also declined to less than 2% of the maximum after 24 h [39]. Plasma concentrations of 500 μM can be reached in mice when metformin is administered by i.p. injection. In this case, metformin further accumulates in liver and kidney and reaches millimolar concentrations. Although the retention in tumors was much lower, the i.p. administration could be a promising approach for tumor patients [40]. In addition, the use of milimolar concentrations of metformin for in vitro assays seems to be relevant, as other nutrients like serine are available in supraphysiological concentrations in the medium that are known to reduce metformin sensitivity [41].

Despite the anticancer effects of metformin, its application as an adjuvant in tumor therapy offers conflicting results. Therefore, we established an in vitro tumor therapy model in order to investigate interactions between metformin and several chemotherapeutic agents. First, the applicability of the new BALB-TTM was proven successfully with four chemotherapeutic agents from different classes. In this case, treatment for 72 h was sufficient to decrease the number of type-III foci significantly even in non-toxic concentrations for Dtx, MMC and 5-FU. Such an effect was observed for Dox only in toxic concentrations. Second, the combined therapy with metformin was tested. An evidence for the cytoprotective role of metformin was given already via the Resazurin assay as metformin could mitigate the cytotoxic effects of Dtx and MMC. In the BALB-TTM, such an chemoresistance-inducing effect was shown for Dox and Dtx. In various in vitro and in vivo studies metformin was shown to decrease Dox-induced cardiotoxicity and is considered as a promising approach for patients treating with Dox [19]. Moreover, metformin could not only reduce the therapeutic concentration of Dox and diminish cardiotoxic side effects, but also shows synergistic anti-tumor effects for prostate [42] and breast cancer [19, 43–47] in different cell and mouse models. However, in the present study metformin could not improve the anticancer effects of Dox in the BALB-TTM. To the contrary, the number of type-III foci increased slightly but not significantly. Consequently, the therapeutic effect seems to be highly dependent on the type of tumor. For metastatic castration-resistant prostate cancer, Dtx is the first-line chemotherapeutic agent. Since the treatment is associated with considerable toxic side effects, there is a need for chemosensitizing agents and it was shown that metformin is able to improve the prognosis [48]. However, in vitro studies with different prostate cancer cell lines treated with metformin and Dtx demonstate controversial results [20, 49]. A clinical study regarding the combined effect of Dtx with metformin in patients with castration-resistant prostate cancer showed that metformin did not act as an chemosensitizer and could not improve prostate cancer specific or overall survival [50]. In our study, metformin even offers reverse results as the therapeutic, foci-reducing effect in the BALB-TTM is mitigated. Taken together, the potential role for metformin in prostate cancer therapy remains controversial and seems to be dependent on many individual factors. Thereby, the BALB-TTM offers a potent tool to elucidate the molecular interactions between Dtx and metformin.

In order to explain our observed effects of the combined therapy with MMC and metformin, we have focused on glucose metabolism. A deregulated energy metabolism in general is characteristic for several tumor cells and especially the glucose metabolism seems to be a promising target for cancer therapy [51]. MMC is a DNA cross linker that requires reductive activation (bioreduction) to exert its chemotherapeutic effects [52]. As mentioned elsewhere [53], an enhanced glycolytic rate results in higher NAD(P)H and thiol levels. Consequently, the induced intracellular reducing environment is able to facilitate the bioreduction of MMC. The effect of metformin on energy metabolism varies highly depending on the cell type and status of transformation. Therefore, we investigated the impact of metformin on glucose consumption and AMPK activation in non-transformed BALB/c fibroblasts first. In line with the observed effect in muscle cells [9] and podocytes [8], metformin increases the glucose consumption in the BALB/c cells dose-dependently. However, even when glucose concentration reaches a minimum of 0.5 g/l, the AMPK becomes not activated. Therefore, metformin seems to impair glucose metabolism in the utilized cell line without affecting the cellular energy sensor AMPK.

Due to the observed increase in glucose consumption, we have expected a synergistic effect of metformin and MMC in the BALB-TTM. Despite the higher glycolytic rate, metformin induced resistance to MMC in our studies. Indeed, a higher glucose consumption after metformin treatment is described only for healthy, peripheral tissue [8, 9]. For cancer cells, a converse effect with lower glucose consumption after metformin treatment was shown that is further described as an inhibition of the Warburg effect [12–16]. Therefore, we measured glucose consumption during the BALB-TTM in non-transformed monolayer cells and in the mixed population with cell foci of malignant transformed cells. As expected, we observed an increased consumption after metformin treatment in non-transformed cells but surprisingly, this was also the case in the mixed population. In fact, the MCA/TPA treated cells show even a higher glucose consumption compared to the non-transformed monolayer cells. At this point, a major limiting factor is the co-existence of non-transformed BALB/c monolayer cells and the malignant transformed, foci forming cells. Thus, we cannot precisely investigate the specific effect of metformin on the malignant transformed cells and have to consider, that the increase in glucose consumption is only due to the non-transformed monolayer cells. Possibly, metformin did not increase glycolysis in malignant transformed cells of the BALB-TTM and therefore did not enhance the therapeutic effect of MMC. In order to clarify the specific effects of metformin on malignant transformed cells in the BALB-TTM, investigations in isolated malignant transformed cells are strongly necessary.

## Conclusion

In conclusion, we have established an in vitro tumor therapy model that offers a helpful tool for investigating molecular mechanisms of tumor therapeutic drugs. In this model, metformin as an adjuvant mitigated the chemotherapeutic effects of Dox and Dtx. Mechanistically, an increase in glucose consumption after metformin treatment was observed but a major limiting factor for clarifying cell specific mechanisms remains the co-existence of non-transformed and malignant transformed cells on the same plate. Finally, this paper indicates a cautious use of metformin during chemotherapy.

## Supplementary Information

**Additional file 1:** **Supplementary Figure 1.** Densitometric analysis of Western Blot results exemplary shown in Fig. 1b. Data are shown as mean + standard error of 3 independent experiments, normalized to α-Tubulin and shown relatively to the 24 h control. Densitometric analysis was performed using the software ImageJ 1.48v.

**Additional file 2:** **Supplementary Figure 2.** Original Western Blots used for Fig. 1b. Proteins were extracted and protein expression as well as phosphorylation levels of AMPK at Thr172 were detected via immunoblot in 3 biological replicates. After detection of p-AMPK, the membrane was stripped two times and re-probed with AMPK mAB and α-Tubulin mAB to confirm equal loading. The red boxes indicate the cropped regions used in the representative figures.

## Data Availability

The datasets used and/or analysed during the current study are available from the corresponding author on reasonable request.

## Abbreviations

5-FU: 5-Fluorouracil
AMPK: 5′ AMP-activated protein kinase
BALB-CTA: BALB/c cell transformation assay
BALB-TTM: BALB/c tumor therapy model
CI: Combination Index
Dox: Doxorubicin
Dtx: Docetaxel
MCA: 3-methylcholanthrene
MMC: Mitomycin C
TPA: 12-tetradecanoylphorbol-13-acetate
