# Supplementary material for: Metformin alters therapeutic effects in the BALB/c tumor therapy model
Source: BMC Cancer. 2021 May 28;21:629. doi: 10.1186/s12885-021-08354-x (PMC8161985; doi:10.1186/s12885-021-08354-x)

Metformin alters therapeutic effects in the BALB/c tumor therapy model

Felix B. Meyer[1], Sophie Goebel[1], Sonja B. Spangel[1], Christiane Leovsky[1], Doerte Hoelzer[1], René Thierbach[1]*

[1]Friedrich-Schiller-Universität Jena, Fakultät für Biowissenschaften, Institut für Ernährungswissenschaften, Abteilung Humanernährung

*Corresponding author with correspondence to rene.thierbach@uni-jena.de

Additional file 1

Additional file 1: Supplementary figure 1.

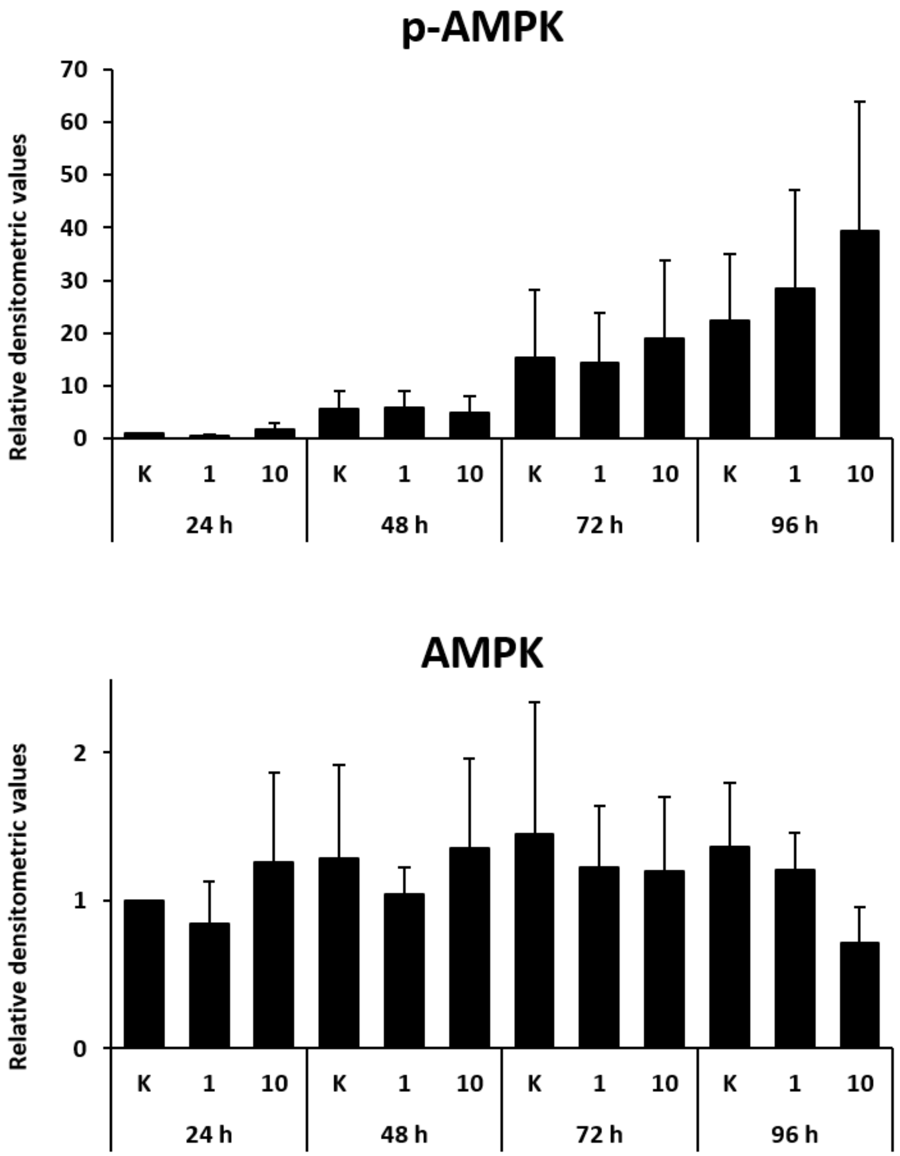

Supplement: Supplementary file 1 — Additional file 1: Supplementary Figure 1. Densitometric analysis of Western Blot results exemplary shown in Fig. 1b. Data are shown as mean + standard error of 3 independent experiments, normalized to α-Tubulin and shown relatively to the 24 h control. Densitometric analysis was performed using the software ImageJ 1.48v. [file 12885_2021_8354_MOESM1_ESM.pdf]
