# Supplementary material for: Metformin alters therapeutic effects in the BALB/c tumor therapy model
Source: BMC Cancer. 2021 May 28;21:629. doi: 10.1186/s12885-021-08354-x (PMC8161985; doi:10.1186/s12885-021-08354-x)

Metformin alters therapeutic effects in the BALB/c tumor therapy model

Felix B. Meyer[1], Sophie Goebel[1], Sonja B. Spangel[1], Christiane Leovsky[1], Doerte Hoelzer[1], René Thierbach[1]*

[1]Friedrich-Schiller-Universität Jena, Fakultät für Biowissenschaften, Institut für Ernährungswissenschaften, Abteilung Humanernährung

*Corresponding author with correspondence to rene.thierbach@uni-jena.de

Additional file 2

Additional file 2: Supplementary figure 2.

p-AMPK (Thr172)

Expected weight: 62 kDa

The red boxes indicate the cropped regions used in the representative figures.

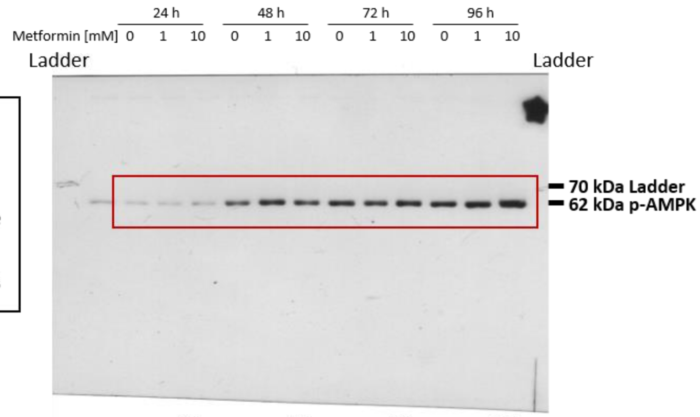

AMPK

Expected weight: 62 kDa

The red boxes indicate the cropped regions used in the representative figures.

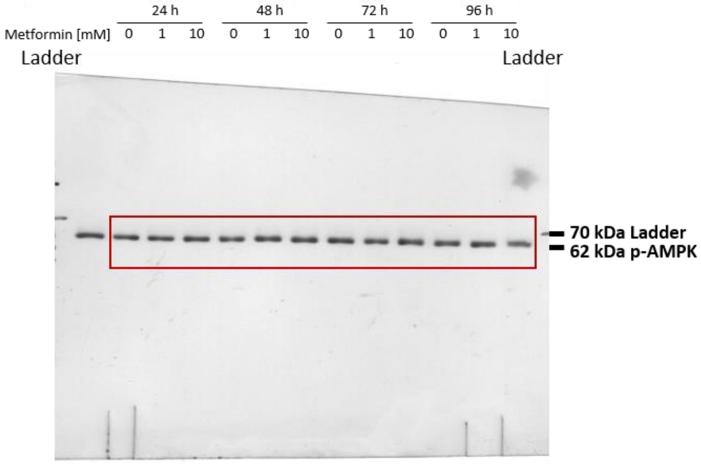

α-Tubulin

Expected weight: 50 kDa

The red boxes indicate the cropped regions used in the representative figures.

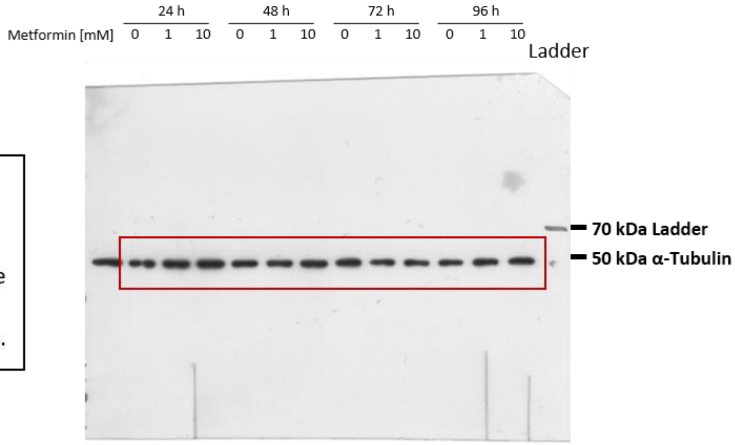

Supplement: Supplementary file 2 — Additional file 2: Supplementary Figure 2. Original Western Blots used for Fig. 1b. Proteins were extracted and protein expression as well as phosphorylation levels of AMPK at Thr172 were detected via immunoblot in 3 biological replicates. After detection of p-AMPK, the membrane was stripped two times and re-probed with AMPK mAB and α-Tubulin mAB to confirm equal loading. The red boxes indicate the cropped regions used in the representative figures. [file 12885_2021_8354_MOESM2_ESM.pdf]
